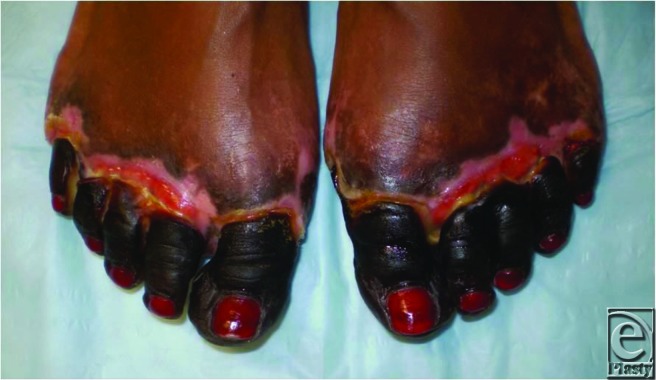# Symmetrical Peripheral Gangrene

**Published:** 2012-07-02

**Authors:** Rudolph A. Cartier, Catherine Tchanque-Fossuo, Malachy E. Asuku, Leigh Ann Price, Stephen M. Milner

**Affiliations:** ^a^College of Osteopathic Medicine, University of New England, Biddeford, ME; ^b^Department of Plastic and Reconstructive Surgery, Johns Hopkins School of Medicine, Baltimore, MD

## DESCRIPTION

A 27-year-old woman presented to the clinic with well-demarcated symmetrical dry gangrene of all the toes. The onset was insidious during recent hospitalization for urosepsis and shock. There is no antecedent history of peripheral ischemia, use of oral contraceptive pill, or smoking. Her medical history is significant for hypertension and preeclampsia. Dorsalis pedis and posterior tibial pulses were palpable.

## QUESTIONS

1. **Discuss the differential diagnoses.**
2. **Discuss the etiopathogenesis of symmetrical peripheral gangrene (SPG).**
3. **What treatment options are available to this patient?**

## DISCUSSION

Dry gangrene is commonly a result of arterial occlusion associated with limited putrefaction and absence of invasive bacterial proliferation. The onset is usually insidious and is dependent upon vascular anatomy; robust blood supply is protective while precarious blood supply served by end arteries increases susceptibility. Commonly affected parts of the body, therefore, include the toes, fingers, penis, ear lobes, feet, and hands. The usual causes of dry gangrene are large vessel diseases as seen in diabetes mellitus, atherosclerosis, and long-term smoking.^1^ Less frequent causes include microvessel angiopathy associated with autoimmune vasculitis and connective tissue diseases such a scleroderma.^2^ Certain infections, trauma, severe burns, and frostbite are known to cause specific types of gangrene.^3^ Iatrogenic gangrene related to inappropriate use of vasoactive drugs such epinephrine and ergot alkaloids have been described.^4^ The caution against use of local anesthetic agents with epinephrine in parts of the body supplied by end arteries has remained in the realm of surgical dogmas and myths.

Symmetrical peripheral gangrene is a rare clinical syndrome characterized by bilateral distal ischemic damage leading to gangrene in the absence of major vascular occlusive disease.^5^^,^^6^ Peripheral pulses are usually palpable as a result of sparing of larger vessels. The mechanism of vascular occlusion is poorly understood though disseminated intravascular coagulation (DIC) has been implicated as the final common pathway in its pathogenesis. Some authors have in fact described it as the cutaneous marker of DIC.^7^ A wide array of infective and noninfective etiological factors has been linked with SPG.^5^ It has been described in conditions associated with sepsis, low-flow states, vasospastic conditions, myeloproliferative disorders, and hyperviscosity syndromes.^6^ The condition is aggravated by hypothermia, vasopressor infusion, immunosuppression, malignancy, diabetes mellitus, and renal failure.^5^^,^^6^ Seasonal variation was recently implied with more cases seen in the winter compared to warmer seasons. Symmetrical peripheral gangrene carries a mortality rate as high as 35% to 40% and an equally high morbidity rate; the literature reports an amputation rate upwards of 70%.^8^^,^^9^

Suggested first-line measures when identified early include discontinuation of vasopressors, reversal of DIC by cautious anticoagulation, and aggressive treatment of shock and sepsis. Adjuvant therapy with tissue plasminogen activator, plasmapheresis, sympathetic blockade, and aspirin has been recognized to contribute to favorable outcome. The patient in question required multiple doses of systemic phenylephrine and norepinephrine infusion to combat hypotension during her recent hospitalization for *Escherichia coli* sepsis following pyelonephritis. This scenario fits the ever-widening etiological spectrum of SPG.^6^ Amputation of the toes remain the only treatment option available to the patient at this point of established gangrene. The level of amputation is however determined by the line of demarcation in concert with considerations of the biomechanics of stump stability, weight bearing, and ambulation.^10^

## Figures and Tables

**Figure:**